# New Publications

**Published:** 1897-01

**Authors:** 


### NEW PUBLICATIONS.

*Diseases of the Dog and their Treatment.* By Dr. GEORGE MULLER, Director of the Clinic for Pet Animals at the Veterinary High School at Dresden. Translated, revised, and augmented by Alexander Glass, A.M., V.S., Lecturer on Canine Pathology at the University of Pennsylvania. Now in press. The book will contain some ninety-three illustrations.

*The Veterinary Blue-book of New York* is in preparation under the auspices of the Veterinary Medical Society of New York County. Edited by the President, Dr. Huidekoper. It will contain a complete list of all the veterinarians of New York City, together with their titles, official positions, and a list of the officers and members of the United States and New York State Veterinary organizations, and many other societies of general interest to all veterinarians and horsemen. It will be the first attempt of this kind of a publication in this country, and will be watched with much interest and we are sure well received.

Professor A. Liautard, of the American Veterinary College, is preparing a work on the *History of American Veterinary Medicine.* Such a work from one so well known to the American veterinary profession, and who has spent so many useful years in the ranks, should prove interesting, valuable, and entertaining.

The Pennsylvania State Veterinary Sanitary Board has issued, under the direction of Dr. Leonard Pearson, one of the most complete and important contributions to the *Study of the Diseases of Poultry* in the English language. The extent and value of this industry to our people are told in figures that are startling to one who has not given the subject consideration. Numerous illustrations of the parasitic diseases, external and internal, add much to the value of this contribution, and it will be much sought after by our people, with grateful appreciation of its author and the board under whose direction it is issued. This department, as well as that of Forestry in Pennsylvania, is doing a public service that must surely allay any fears or misgivings of those who doubted the wisdom of establishing these new departments of our State government.

A third edition of Walley's *Guide to Meat-inspection* is announced, it having been completed by Professor McFadyean owing to the death of Professor Walley.

---

Dr. James Robertson, in a paper before the Illinois State Veterinary Medical Association, in November, on "The Relation of the Veterinary Profession to Horseshoeing," among many other good thoughts, well says: "The veterinarian and the shoer work in harmony. To be able to do so they must understand their respective duties. The veterinarian should have a practical knowledge of shoeing and be able to level and balance a foot, perform all operations on a foot, do his own surgery to suspected injuries, nail-pricks, suppurated corns, etc. The shoer, in addition to his mechanical skill, should know the structure of the machine he is expected to keep in repair, so that he may be able to assist a defective foot, adjusting the bearings for the proper workings of the joints, enabling the ligaments to keep the bone more accurately in position and the tendons to perform their functions of flexion and extension."

